# Artificial cranial deformation in Tiwanaku, Bolivia

**DOI:** 10.1007/s00381-023-06094-w

**Published:** 2023-08-18

**Authors:** Pranay Narang, Zain Jandial, Jorge Daniel Brun Aramayo, John Crawford, Michael L. Levy

**Affiliations:** 1 University of California, San Francisco, CA USA; 2 University of California, Berkeley, CA USA; 3 Hospital Del Nino, La Paz, Bolivia; 4 Children’s Hospital of Orange County, Orange, CA USA; 5 Division of Pediatric Neurosurgery, Rady Children’s Hospital-San Diego, San Diego, CA USA

**Keywords:** Artificial cranial deformation, Alteration of craniofacial skeleton, Tiwanaku region, Andean civilization, Pre-Columbian Andes

## Abstract

**Purpose:**

Herein lies a brief historical review of the practice of artificial cranial deformation (ACD) in Tiwanaku, Bolivia, a pre-Columbian archeological ruin once regarded as one of the most powerful pre-Inca regions whose influence extended into present-day Peru and Chile from 600 to 1000 AD. We describe the history, purpose, and implications of ACD from both a neuroanatomical and cultural perspective.

**Methods:**

A literature review was conducted through PubMed on the history of artificial cranial deformation in South America, concentrating on the Tiwanaku region. The authors searched all available data with no specific time reference, using the mentioned keywords: ACD, neuroanatomical implications of ACD, cultural and social functions of ACD, Tiwanaku society, and Andean civilization.

**Results:**

Early Andean civilization was hierarchical and stratified. In Tiwanaku, the practice of ACD served to delineate one’s social class, caste, lineage, and vocation. This was especially useful for warriors, who distinguished their fellow combatants from insurgents by differences in their cranial structure. ACD was usually conducted within the first few months of an infant’s life before morphogenetic features became permanent. Two popular cranial styles—*tabular* and *annular*—were achieved by applying various mechanical apparatus and resulted in several cranial shapes (conical, box-like, flattened, etc.). Neuroanatomically, each deformation technique and the duration for which mechanical stress was applied influenced the solidification of cranial bones and shaped the frontal, occipital, parietal, and temporal bones differently. Cognitive deficits and plagiocephalic defects were recorded in limitation and may have been overlooked as the era’s occupational demands were more labor-intensive than knowledge-driven.

**Conclusion:**

In Tiwanaku, the custom of ACD was used to demonstrate group identity, with alterations of the cranial shape corresponding to a particular headdress. ACD was used to distinguish an individual’s social identity, separating different groups of society into castes, classes, and slaves (Brain, 1979). The custom has also been used to mark territory and emphasize ethnic differences among groups, with potential cognitive implications that were largely unrecorded.

## Introduction

For millennia, the practice of modifying the human body—between tattooing, piercing, circumcision, clitoridectomy, foot binding, and facial scarification—has been customary for a range of cultural, religious, and social purposes. Of these, few have been as widespread and long-lasting as artificial cranial deformation (ACD), the practice of intentionally molding an infant’s skull into a particular shape. This was carried out by applying a prolonged, low-intensity compressive force to a newborn’s skull from the first days of life until 2–4 years of age through belts, bands, cords, boards, flat surfaces, and other mechanical apparatus [1, 2].

While contemporary audiences may view ACD as inhumane, it was central to many cultures and societies spanning nearly every continent. Not only was it common in present-day Malaysia, Indonesia, Sumatra, Borneo, the Philippines, India, Pakistan, Afghanistan, Turkmenistan, and Baluchistan but the earliest evidence dates back to 45,000 BC when Neanderthal remains were recovered from Shanidar Cave in Iraq [3, 4]. Interestingly, the earliest written record of ACD is also credited to Hippocrates in 400 BC. In his description of the *Macrocephali*, or long heads, named for their use of this custom, Hippocrates astutely noted that cranial elongation may be associated with high social stature, identified that infants’ heads were fashioned by human hands, and suggested, in accordance with his supposed belief in the inheritance of acquired characteristics, that elongated skulls may be passed on to future generations [5]:

"There is no other race of men which have heads in the least resembling theirs… they think those the most noble who have the longest heads… immediately after the child is born, and while its head is still tender, they fashion it with their hands, and constrain it to assume a lengthened shape by applying bandages and other suitable contrivances whereby the spherical form of the head is destroyed, and it is made to increase in length… If, then, children with bald heads are born to parents with bald heads; and children with blue eyes to parents who have blue eyes; and if the children of parents having distorted eyes squint also for the most part; and if the same may be said of other forms of the body, what is to prevent it from happening that a child with a long head should be produced by a parent having a long head? Text A: part 14, on airs, waters, and places, the genuine works of Hippocrates [5, 6]"

Today, artificially deformed skulls have been discovered all over Europe, between Romania, Italy, Belgium, France, and the UK, and the whole of the American continent, between North America and Patagonia [3]. In the Americas, cranial deformation is described to have been most widespread in the Andean region. There, ACD served a range of cultural functions and was conducted through several techniques that culminated in various aesthetics.

The full scope of this cultural and technical diversity is yet to be thoroughly understood, and it is still debated whether there were any neuroanatomical or cognitive consequences of ACD. The aim of this brief historical review is twofold: firstly, to elucidate the function of ACD in Tiwanaku, Bolivia, one of the longest-lived South American pre-Columbian polities whose cultural influence extended into Peru and Chile; and secondly, to explore possible craniofacial, neurological, and neuroanatomical implications of each deformation technique popularized in Tiwanaku. Given that this practice can only be examined through archeological relics, conclusions can only be inferred and are tenuous at best. It is our hope that this discussion will renew interest and investigation into an ancient and universal practice that was once central to mankind’s earliest societies.

## ACD in the Americas: brief review

It has been suggested that ACD reached the Western Hemisphere when America’s first ancestors crossed the Bering Strait, arrived along the Alaskan coasts, and migrated southwards. Until 1000 AD, ACD was common among the Apache, Mohave, Yuma, and Pueblo Indians in the southwestern United States. It has also been traced east of the Mississippi, extending to the Atlantic Ocean, but it has not been documented among the Native Americans of the Great Plains region [7]. ACD has also been described in Mexico, Nicaragua, Costa Rica, Guatemala, Jamaica, Cuba, and Puerto Rico, particularly among the Mayans [8], with some evidence dating back 2000 years.

In South America, ACD was widespread throughout the Andean region, from Venezuela to Guyana, Colombia, Ecuador, Peru, Bolivia, Chile, and Argentina, though it is absent from the Brazilian Amazon [9]. While the origin of ACD is oftentimes mistakenly attributed to the Incas, the ancient Peruvians practiced cranial deformation for at least 1,000 years prior to the assembly of the Inca State.

## Technical and cultural diversity of ACD in Tiwanaku, Bolivia

The Tiwanaku society flourished in the south-central Andes from 500 to 1150 AD. It hosted 40,000 inhabitants, expansive irrigation networks, robust monumental architecture, erected monoliths and was characterized by settlement patterns that suggested social hierarchy and stratification. It has been recognized by archeologists as an influential pre-Inca force and the political core of the region [10–12]. Situated on the borders of present-day Bolivia, Peru, and Chile, its favorable geographic position enabled rituals such as ACD to be diffused into different lands, moieties, and ethnic groups.

From a technical perspective, the deformation style and instruments utilized varied by region, with the tabular style predominating the Moquegua Valley, or Southwest Tiwanaku, and the annular style predominating the Katari Valley, or Northeast Tiwanaku. Between these borders, both styles—and the resulting flattened and conical head shapes—coexisted among an ethnically diverse population (Table 1).

It should be noted that there may have been cases of unintentional cranial modifications, particularly owing to usage of the cradleboard. It has been suggested that the cradleboard may have served as a transportation device intended solely to limit infant mobility. With repeated usage, the compressive force may have inadvertently resulted in cranial changes.

From a cultural and social perspective, ACD was primarily used in Tiwanaku to distinguish group identities. With cranial alterations corresponding to specific headdresses, ACD visibly stratified the population into castes, classes, and slaves. It was also practiced to mark territory and emphasize ethnic differences between groups. Motives may also have included the demonstration of one’s socioeconomic status, supposed health benefits, and greater attractiveness [13].

**Table 1:** Deformation Techniques and Cranial Alterations

**Deformation style**	**Cranial shape**	**Cranial bones altered**	**Tiwanaku region of concentration**
**Tabular oblique deformation:** two wooden boards (oftentimes padded) were placed across the frontal and occipital regions of infants’ heads and fastened via bandages. Two compressive forces were applied to the skull anteriorly and posteriorly, with the latter force centered at the inion and exerted between the bottom of the occipital bone and the lambdoid suture	Box-like, flattened cranial vault. Cranium may have appeared bilobular, with a lateral bulging of the head [2]. Cranial base may appear wider and shallower, and the face may be foreshortened and wider [16]	Frontal and occipital bones were flattened. Postero-lateral parietal bones were widened. Sinuous skull contour may have been created from padding [2, 19]	Moquegua Valley, or Southwest Tiwanaku
**Tabular erect deformation:** infants’ bodies were dorsally laid upon a flat, wooden surface. A vertical section of wood was bandaged to the flat, wooden surface at an angle, projecting upwards and resting upon the frontal region of the infants’ skulls. Infants were fastened within both wooden sections, known as a *cradleboard*. The angle between both wooden surfaces and the tightness of the bandage determined the overall compressive force	Box-like, flattened cranial vault. Cranium may have appeared bilobular, with a lateral bulging of the head [2]. Cranial base may appear wider and shallower, and the face may be foreshortened and wider [16]	Frontal and occipital bones were flattened, but shape depended on angle and tightness of the cradleboard’s surfaces. Postero-lateral parietal bones were widened. [2, 19]	Moquegua Valley; Southwest Tiwanaku
**Annular style:** bands, belts, and wrappings were transversely strapped around infants’ heads with varying tightness. The compressive force was applied to the skull circumferentially (Fig. 1)	Conical cranial vault	Posterior parietals extended superiorly and posteriorly; bones of the frontal, occipital, and cranial base lengthened [2, 19]	Pampa Koani and Lukurmata in Katari Valley; Northeast Tiwanaku

**Fig. 1:**
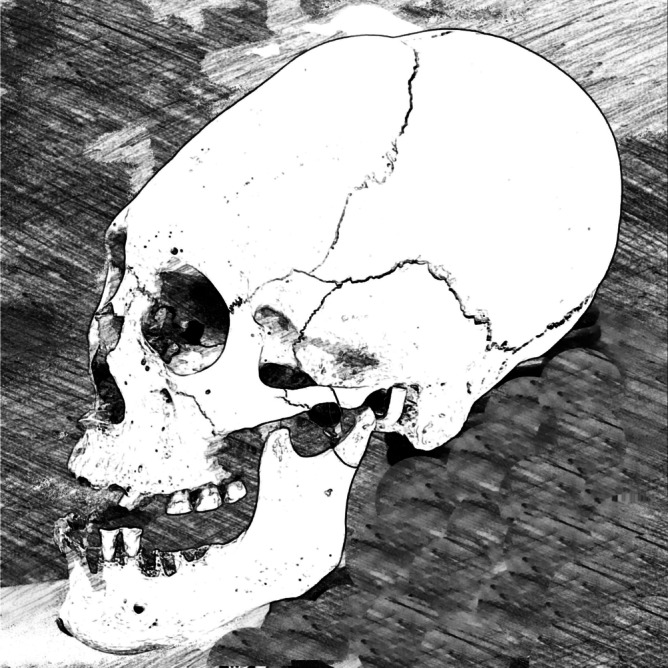
Illustration of annular cranial deformation style. The conical cranial vault is derived by wrapping bands, belts, and wrappings transversely around infants’ heads, resulting in a compressive force applied circumferentially on the skull

The most deformed skulls that have been discovered from the early Bolivian region were those of women. It is believed that, for women, ACD served an aesthetic and social function, in which certain cranial alterations were made to instill specific personality characteristics, such as protection from demons and spirits [14]. Alternatively, in some tribes, particularly the Aymaras of Bolivia, the Caribe Indians in Columbia, and the Patagones in Argentina, cranial deformation was primarily performed on men to signify membership within the community’s “warrior class.” This enabled soldiers to discriminate their fellow combatants from insurgents and invaders, facilitating recognition and protection.

It should also be noted that while anthropologists and historians concur that elongated skulls are products of ACD, in recent decades, alternative theories have gained traction, purporting that the atypical shape of these skulls is evidence of extraterrestrial intervention. The Museo Ritos Andinos (Andean Rites Museum), situated in the Peruvian province of Quispicanchi, east of Cusco, has organized various exhibitions showcasing such “alien skeletons.”

## Review of proposed craniofacial, neuroanatomical, and neurological implications

At the time of this writing, bioarcheological and statistical analyses indicate that cranial deformation may have impacted the entire craniofacial skeleton, including facial height, nasal height, orbital height, frontal breadth, orbital breadth, bizygomatic breadth, and maxilla-alveolar breadth [15–18]. This impact was especially pronounced among components of the cranial skeleton that articulate with the occipital bone, including lambdoid ossicles and occipitomastoid intrasuturial bone [13]. In fact, skulls of pre-Columbian mummies reveal an increased presence of posterior and lateral Wormian bones, exostosis of the outer ear canal, and persistence of the metopic suture [3].

While cognitive deficits associated with traumatic brain injuries and accidents involving rapid, hard impacts cannot definitively be extended to ACD given the gradual, graded nature of the aforementioned deformation techniques, such impairments may have manifested to varying degrees. While any proposed effects are tenuous and inconclusive, damage to the frontal lobe, as characteristically evidenced in patients suffering from brain damage, may have impaired one’s memory, attentiveness, executive functioning, processing speed, and behavior. Likewise, damage to the parietal lobe may have impaired one’s ability to discriminate between sensory stimuli; interpret auditory, visual, motor, and sensory signals; translate sensory information into memory; and recognize parts of the body or writing; damage to the temporal lobe may have resulted in irritability, hearing deficits, and impaired verbal memory and interpretation of others’ emotions; and damage to the occipital lobe may have compromised one’s visual reception and interpretation of colors, shapes, and objects [2]. Some scholars also suggest that, in circumstances where pressure was applied to the hippocampus during deformation, soon after, or later in an infant’s development, ACD may have resulted in epileptic seizures [13]. It is possible that such cognitive impairments were overlooked given that occupational demands were more labor-intensive than knowledge-driven.
